# The Claustrum in Relation to Seizures and Electrical Stimulation

**DOI:** 10.3389/fnana.2019.00008

**Published:** 2019-02-12

**Authors:** Lalitha Kurada, Arezou Bayat, Sweta Joshi, Mohamad Z. Koubeissi

**Affiliations:** Department of Neurology, The George Washington University, Washington, DC, United States

**Keywords:** attention, dyscognitive seizures, kainic acid, connectivity, electrical stimulation

## Abstract

The neural mechanisms of altered consciousness that accompanies most epileptic seizures are not known. We have reported alteration of consciousness resulting from electrical stimulation of the claustrum *via* a depth electrode in a woman with refractory focal epilepsy. Additionally, there are reports that suggest possible claustral involvement in focal epilepsy, including MRI findings of bilaterally increased T2 signal intensity in patients with status epilepticus (SE). Although its cytoarchitecture and connectivity have been studied extensively, the precise role of the claustrum in consciousness processing, and, thus, its contribution to the semiology of dyscognitive seizures are still elusive. To investigate the role of the claustrum in rats, we studied the effect of high-frequency stimulation (HFS) of the claustrum on performance in the operant chamber. We also studied the inter-claustral and the claustro-hippocampal connectivity through cerebro-cerebral evoked potentials (CCEPs), and investigated the involvement of the claustrum in kainate (KA)-induced seizures. We found that HFS of the claustrum decreased the performance in the operant task in a manner that was proportional to the current intensity used. In this article, we present previously unpublished data about the effect of stimulating extra-claustral regions in the operant chamber task as a control experiment. In these animals, stimulation of the corpus callosum, the largest interhemispheric commissure, as well as the orbitofrontal cortex in the vicinity of the claustrum did not produce that same effect as with claustral stimulation. Additionally, CCEPs established the presence of effective connectivity between both claustra, as well as between the claustrum and bilateral hippocampi indicating that these connections may be part of the circuitry involved in alteration of consciousness in limbic seizures. Lastly, some seizures induced by KA injections showed an early involvement of the claustrum with later propagation to the hippocampi. Further work is needed to clarify the exact role of the claustrum in mediating alteration of consciousness during epileptic seizures.

## Introduction

The neural correlates of consciousness are not fully understood. Altered consciousness is the hallmark of focal dyscognitive seizures, which are characterized by loss of perception of external and internal stimuli during wakefulness (Blumenfeld, 2011). However, the precise brain structures and mechanisms involved in the pathophysiology of ictal impairment of consciousness are yet to be identified (Arthuis et al., 2009; Bartolomei and Naccache, 2011; Blumenfeld, 2012). Regardless of the seizure onset zone and variation in semiology, the claustrum (Koubeissi et al., 2014) as well as the frontoparietal association cortex and the subcortical arousal system in the brainstem and thalamus (Blumenfeld, 2012), have been implicated in ictal alteration of consciousness. While recent work has investigated the role of the claustrum in certain complex behaviors (Smith and Alloway, 2010; Wang et al., 2017; White et al., 2017), attention (Mathur, 2014; Goll et al., 2015), and salience processing (Remedios et al., 2010, 2014) the main focus of this review is on the role of the claustrum in consciousness as seen in epilepsy and electrical stimulation.

## Neuroanatomical Architecture of the Claustrum

A detailed up-to-date description of the anatomy is beyond the scope of our report, which is mostly concerned with epilepsy and electrical stimulation of the claustrum. The anatomical aspects and nomenclature are detailed by others (Johnson and Fenske, 2014; Mathur, 2014). However, we review specific neuroanatomical aspects here. The claustrum was originally termed as “nucleus taeniaformis” by Vicq d’Azyr, and later renamed as “claustrum” by Burdach (Rae, 1954). The claustrum (literally, “hidden away”) is a highly conserved, thin, irregular sheet of gray matter, curved and embedded in the white matter of the cerebral hemispheres beneath the neocortex. The exact shape of the claustrum varies among different species. In humans, it occupies only 0.25% of the total volume of the cerebral cortex (Kowianski et al., 1999), and is situated between the putamen and the insular cortex, separated from these structures by the extreme capsule laterally and the external capsule medially. The ventral claustrum is fragmented by fiber bundles related to the anterior commissure and the uncinate fasciculus and extends laterally to the amygdaloid complex.

### Cell Types

The claustrum has strikingly few neuronal types compared with those of the cerebral cortex. Two common cell types can be distinguished. The first are medium to large, spiny stellate, or fusiform cells (Brand, 1981; LeVay and Sherk, 1981; Braak and Braak, 1982) which are the common cell types. These spiny cells possess long, coarse axons, often leaving the claustrum either laterally or medially. They send and receive projections to and from the cerebral cortex and their dendrites do not have a preferred orientation. They have varied soma shapes, including pyramidal, fusiform, and spherical. The second type of claustral cells are the small, granular, spine-free cells with axons forming dense local arborizations. The characteristic feature of these small cells is that the axons do not leave the claustrum and are GABAergic. These cells can be subdivided histochemically by the presence of different neuropeptides or calcium-binding proteins. The potential implication of the paucity of claustral cell types in synchronizing the perception of a stimulus across multiple primary sensory cortices has been discussed elsewhere (Crick and Koch, 2005).

### Claustrum Connectivity

The claustrum is the most densely connected structure by volume in the human brain (Torgerson et al., 2015). The claustrum can be divided into three sub-regions according to different cortical connections: (1) the anterior-dorsal region connected to the somatosensory and motor cortices; (2) the posterior dorsal region connected to the visual cortex; and (3) the ventral region connected to the auditory cortex (Baizer et al., 2014; Mathur, 2014; Milardi et al., 2015). Identification of the anatomical boundaries of the rodent claustrum, as well as its connectivity with various brain regions has been methodologically difficult due to the poor delineation of the extreme capsule resulting in a continuous structure of the claustrum with neighboring cortices (Mathur et al., 2009; White et al., 2017). Recent studies have identified numerous genes that are highly expressed in the claustrum such as Gng2, parvalbumin (Mathur et al., 2009), Gnb4 (Wang et al., 2017). Although not exclusively found in the claustrum, these genes could help delineate its boundaries.

Anatomic connectivity studies across species, including mammals ranging from rodents to primates, have revealed that the claustrum forms extensive reciprocal connections with the allo- and neo-cortical regions including the frontal, premotor, ventral anterior cingulate, hippocampus, entorhinal cortex, temporal, occipital, sensory and motor regions, as well as sub-cortical structures such as the thalamus, basal ganglia, caudate nucleus, putamen, globus pallidus, and lateral amygdala (Pearson et al., 1982; Fernández-Miranda et al., 2008; Milardi et al., 2015; Torgerson et al., 2015; Arrigo et al., 2017; Atlan et al., 2017; Reser et al., 2017; Wang et al., 2017; White et al., 2017). Thus the claustrum might be involved in multimodel integration of sensory information into single conscious percept (Crick and Koch, 2005). Bidirectional connections of some cortical areas with the claustrum have been identified in rats (White et al., 2017), and mice (Wang et al., 2017). Unlike primary somatomotor cortices, the anterior cingulate cortex (ACC; White et al., 2017) and anterior insular cortex (aINS) in rats (Sinai et al., 2005; Mathur et al., 2009; Menon and Uddin, 2010; Remedios et al., 2010; Mathur, 2014) are extensively and bidirectionally connected with the claustrum. Indeed, a recent study using adeno-associated virus in the mouse found sparse connections between the claustrum to the aINS, but extensive reciprocal projections from the aINS to the claustrum (White et al., 2018). In contrast, no such connections were observed in cats (Markowitsch et al., 1984). The endopiriform nucleus, which is implicated in epileptic seizures of temporal and extratemporal origin (Laufs et al., 2011; Vismer et al., 2015) is also connected to the dorsal claustrum in both rats and rabbits (Lipowska et al., 2000). The claustrum and the endopiriform nucleus have been considered separate entities in rodents, they constitute a single continuous structure in primates (Smith et al., 2018). The extensive connectome of the claustrum thus supports the possible role of claustrum involvement in integrating multiple inputs of a single conscious percept (Crick and Koch, 2005).

## Functions of the Claustrum

Direct functional analysis by selective lesion or activation studies has been a challenging to identify the functional aspects of the claustrum, due to its location and structure. Crick and Koch suggested a role of the claustrum in binding the conscious percepts and unifying them in what the individual perceives as a single experience (Crick and Koch, 2005). Further studies are required to understand the claustral involvement in consciousness in synchronizing electrical activity across these widely-distributed cortical networks (Reardon, 2017). Additionally, a number of hypotheses have been proposed that were based on the extensive reciprocal connectivity of the claustrum with numerous brain regions (Remedios et al., 2010; Smith and Alloway, 2010; Smythies et al., 2012; Mathur, 2014; Patru and Reser, 2015). However, the precise functions of the claustrum, including its role and relevance in ictal manifestations of dyscognitive seizures are yet to be explored.

**Figure 1:**
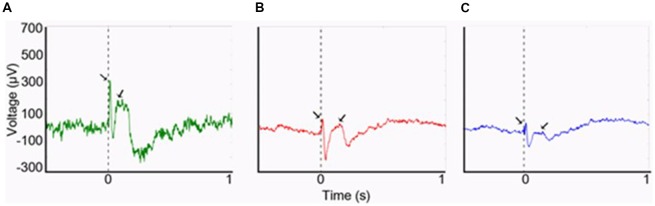
Cerebro-cerebral evoked potentials (CCEPs) obtained by stimulating the left claustrum at 800 μA (0.1 Hz, 500 μs). **(A)** The right claustral CCEP with first negative (N1) peak latency of 12 ms and amplitude of 328 μA. **(B)** Left hippocampal response with a smaller amplitude of N1 (91 μA), though with same N1 latency at 12 ms. **(C)** Right hippocampal response (N1 peak: 85 μA; latency: 16 ms). From Bayat et al. (2018)—with permission.

In humans, at least three separate studies that combined electroencephalography (EEG) with functional MRI (fMRI) assessing interictal epileptiform discharges in individuals with focal epilepsy found increased blood-oxygen-level-dependent (BOLD) signal in the piriform area in association with spikes (Laufs et al., 2011; Fahoum et al., 2012; Flanagan et al., 2014; Vaughan and Jackson, 2014). Indeed, based on the spatial resolution of fMRI, activation of the claustrum may be indistinguishable from that of the piriform cortex. As part of an investigation of the possible role of the claustrum in mediating the semiology of temporal lobe epilepsy, we have demonstrated the presence of a robust connectivity between the claustra and the hippocampi using cerebro-cerebral evoked potentials (CCEPs; Bayat et al., 2018; Figure 1). These results constituted an *in vivo* evidence of known connections that may be part of the circuitry involved in alteration of awareness in limbic seizures. Importantly, seizure-related inhibition of thalamic and brainstem structures with consequent deactivation of the cortex has been suggested as the neural basis of altered consciousness (Blumenfeld, 2012). The claustrum may be an important node in this process, as suggested by the study by Smith et al. (2017) that utilized seed-based resting-state fMRI (RS-fMRI) and neuroanatomical tracing to study the anatomical connections of the claustrum in relation to its functional connectivity in quiet-awake vs. isoflurane-induced anesthetized rats. During wakefulness, strong claustral interhemispheric functional connections with the medial prefrontal cortex (mPFC) and mediodorsal (MD) thalamus as well as with other cortical areas were seen. In anesthetized rats, however, functional connections with the mPFC and MD-thalamus were attenuated with no significant changes of connections with the rest of the cortex. These suggest that claustral connections with the thalamus and mPFC may be important for arousal.

### Clinical Observations Demonstrating the Roles of Claustrum

A number of clinical studies have reported seizure-induced claustral changes. Sperner et al. (1996) described a 12-year-old girl with a sudden onset of status epilepticus (SE) followed by recurrent focal dyscognitive and myoclonic seizures and psychosis. A brain MRI done within 7 days of seizure onset showed increased T2 signal and decreased T1 signal of both claustra. These changes disappeared on repeat imaging 5 weeks later. In a case study of fatal SE of unknown origin, a 35-year old man with no prior medical history developed seizures 4 days after the onset of a mild flu-like symptoms, but no evidence of viral encephalitis. A motor seizure affecting both arms was followed by loss of consciousness and confusion. On day 9 there was an increase in seizure frequency with a decrease in consciousness. Radiological and neuropathological studies suggested acute bilateral lesions in both the hippocampus and claustrum. While the first scan was normal, the second scan showed high signal lesions on T2 weighed images in the medial aspects of both temporal lobes and right claustrum. Severe neuronal loss was found in the hippocampus and the claustrum (Nixon et al., 2001). Similarly, Ishii et al. (2011) described transient bilateral symmetric claustral changes in the setting of mumps encephalitis in a 21-year-old man presenting as a generalized tonic-clonic seizure. Later, a larger series found six patients among 155 refractory SE cases who had reversible bilateral claustral T2 hyperintensity on MRI, without restricted diffusion. In these patients, confusion, stupor, and acute repetitive seizures with focal motor and myoclonic semiology were common (Meletti et al., 2015).

### Electrical Stimulation Studies

Gabor and Peele (1964) reported that electrical stimulation of the claustrum in non-anesthetized cats resulted, among other symptoms, in altered awareness manifesting as crouching, eye closure, and unresponsiveness to external stimulation. Other studies found that electrical stimulation of the claustrum in cats showed contradicting results of either excitation or inhibition of the cortical neurons causing brief neuronal fast burst, typically followed by a prolonged suppression in both the oculomotor frontal eye field (Salerno et al., 1984; Cortimiglia et al., 1991) and primary visual cortex (Ptito and Lassonde, 1981; Tsumoto and Suda, 1982). These effects could possibly be attributed to the feedforward inhibitory loop originated in the claustrum (Bruno, 2011).

**Figure 2:**
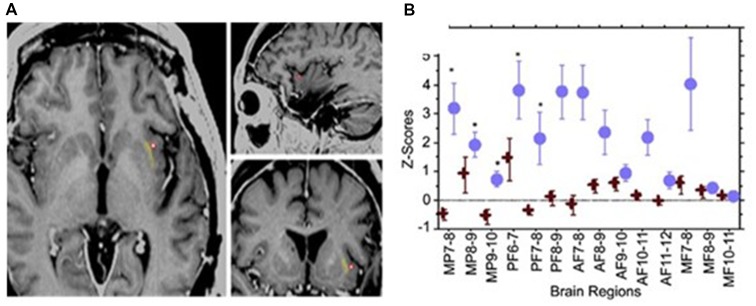
Electrical stimulation of the left claustrum in a patient with intractable epilepsy. **(A)** AI4 contact represented as a red circle, when stimulated elicited impairment of consciousness. **(B)** Representation of 15 selected bipolar channels. Z-scores are used for estimating the variations of h2 coefficients relative to the prestimulation period. Blue circle: two randomly chosen AI4 stimulations. One causes disruption of consciousness. Red cross: two randomly chosen AI4 stimulations that did not interfere with consciousness at lower current intensities. Significant variations are shown in medial parietal (MP) channels and posterior frontal (PF) channels. AF, anterior frontal; MF, medial frontal. From Koubeissi et al. (2014)—with permission.

In a case report (Koubeissi et al., 2014), the left claustrum was electrically stimulated in a 54-year-old woman with intractable epilepsy, with resulting impaired consciousness in a reproducible way. Stimulating the claustrum using biphasic waves at 14 mA (50 Hz, 0.2-ms pulse width, 3- to 10-s train durations), but not at lower current intensities, produced consistent findings (Figure 2). Impairment of consciousness was described as unresponsiveness to visual and auditory commands, blank staring, and an arrest of reading. Cessation of stimulation resulted in an immediate return to baseline with no recollection of the stimulation period; any words given to the patient during stimulation could not be recalled. The patient resumed reading from the same sentence and did not suffer any aphasia or dysarthria. Neither the stereo- nor surface-EEG showed any abnormal discharges that outlasted the stimulation. The stimulation was repeated the next day with consistent findings and stimulation of adjacent electrode contacts did not elicit similar responses. In contrast, no sensorimotor or cognitive impairment was reported in patients where surgical removal of unilateral claustrum for low-grade cerebral glioma (Duffau et al., 2007) or lesions of the claustrum (Chau et al., 2015) Also, claustral lesions associated with striatal lesions do not necessarily cause lack of consciousness (Straussberg et al., 2002).

## Role of the Claustrum in Seizures

The claustrum has been implicated in the generation and maintenance of seizures in kindling models (Wada and Tsuchimochi, 1997; Mohapel et al., 2001; Zhang et al., 2001; Sheerin et al., 2004). Furthermore, employing kindling antagonism of the claustrum blocked amygdala kindling suggesting its stronger access to the seizure motor substrates (Mohapel and Corcoran, 1996). Neuropathological abnormalities have been reported in kainate (KA) model of mature rats (Nitecka et al., 1984) and epileptic beagles (Montgomery and Lee, 1983).

**Figure 3:**
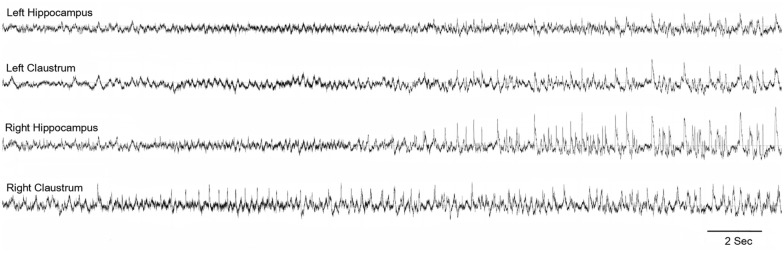
Claustrum as the seizure onset zone: representative electroencephalography (EEG) indicating the onset of seizure in the right claustrum and propagating to the bilateral hippocampi. From Connell et al. (2017)—with permission.

In an attempt to identify the extra-hippocampal seizure onset zones in the intraperitoneal KA model of epilepsy, we have examined the electrographic seizure onsets during the acute and pre-SE seizures and later spontaneous recurrent seizures (SRS; Connell et al., 2017). Preceding and simultaneous ictal activity was observed in multiple locations, including the claustrum, which eventually involved the hippocampus (Figure 3). These findings indicate the early involvement of the claustrum and possibly suggest a role in the initiation and propagation of seizures in this model.

**Figure 4:**
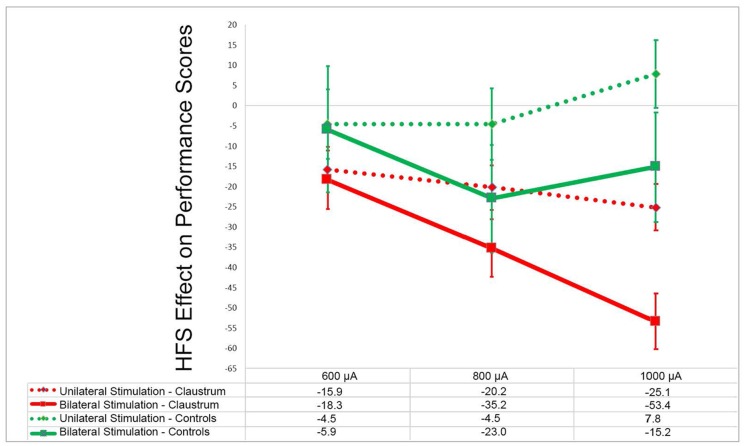
Effects of claustrum stimulation on rat performance in the operant chamber: in the above plot x- and y-axes represent the stimulation current intensity and percentage points of effect size, respectively. In experimental group the electrodes were installed in bilateral claustra. In control group the electrodes were placed in corpus callosum, orbital cortex and frontal cortex (unpublished). GEE analysis in experimental group showed that there was significant decline in the performance score in unilateral and bilateral stimulation at 600–1,000 μA compared with the rest sessions score (*p* < 0.001). In the control group (unpublished) there was not any significant decline in performance at these current intensities in comparison to the rest session (*p* > 0.05). Modified from (Bayat et al., 2018)—with permission.

In an attempt to investigate whether claustral involvement by seizure discharges results in alteration of awareness, we performed an electrical stimulation study of the claustrum in rats while performing the operant conditioning task (Bayat et al., 2018). Occasional behavioral changes such as motor responses, inactivity, and decreased responsiveness were observed. However, even when such behavioral alterations were not obvious, a decreased performance in the operant task, which requires sustained attention, was observed in a manner that was proportional to the current intensity used (Figure 4). Furthermore, in the control group stimulation of the extra-claustral regions such as the corpus callosum and the orbitofrontal cortices that are close to the claustra did not elicit significant reduction in the scores compared to those of the claustral stimulation (not published previously).

## Conclusions and Future Directions

It is important to note that indirect clinical evidence implicating a role for the claustrum in consciousness (such as imaging findings in some patients with SE), and the direct clinical evidence through electrical stimulation, as well as the animal data we have collected so far all continue to be insufficient to formulate a solid hypothesis about the function of the claustrum. Therefore, more controlled experiments in animals and prospective data in humans need to be collected before a clearer picture about the function of the claustrum can be attained.

Also, the KA model focuses on focal epilepsy and thus studies using a wide-range of animal models as well as a genetic model for generalized epilepsy are required to extend the generalizability of the findings. Future studies should also focus on the selection of different stimulation parameters and assessing whether low-frequency stimulation of the claustrum can have anti-seizure effects. With the advancement of direct access to the claustrum using modern techniques such as optogenetics (Wang et al., 2017), precise stimulation studies are possible. Thus the claustrum could be an attractive new target for epilepsy therapy.

## Author Contributions

MK outlined the manuscript and all authors contributed equally. LK generated the first draft.

## Conflict of Interest Statement

The authors declare that the research was conducted in the absence of any commercial or financial relationships that could be construed as a potential conflict of interest.

## Abbreviations

GABA: γ amino butyric acid
TLE: temporal lobe epilepsy
DBS: deep brain stimulation
mPFC: medial prefrontal cortex
MD: mediodorsal
CCEPs: cerebro-cerebral evoked potentials
LFS: low-frequency stimulation
HFS: high-frequency stimulation
RSE: refractory status epilepticus
EEG: electroencephalography
SRS: spontaneous recurrent seizures.
